# T cell response to intact SARS-CoV-2 includes coronavirus cross-reactive and variant-specific components 

**DOI:** 10.1172/jci.insight.158126

**Published:** 2022-03-22

**Authors:** Lichen Jing, Xia Wu, Maxwell P. Krist, Tien-Ying Hsiang, Victoria L. Campbell, Christopher L. McClurkan, Sydney M. Favors, Lawrence A. Hemingway, Charmie Godornes, Denise Q. Tong, Stacy Selke, Angela C. LeClair, Chu-Woo Pyo, Daniel E. Geraghty, Kerry J. Laing, Anna Wald, Michael Gale, David M. Koelle

**Affiliations:** 1 Department of Medicine,; 2 Department of Immunology, and; 3 Department of Laboratory Medicine and Pathology, University of Washington, Seattle, Washington, USA.; 4 Clinical Research Division, Fred Hutchinson Cancer Research Center, Seattle, Washington, USA.; 5 Department of Epidemiology, University of Washington, Seattle, Washington, USA.; 6 Vaccine and Infectious Diseases Division, Fred Hutchinson Cancer Research Center, Seattle, Washington, USA.; 7 Center for Innate Immunity of Immune Disease, Department of Immunology, and; 8 Department of Global Health, University of Washington, Seattle, Washington, USA.; 9 Benaroya Research Institute, Seattle, Washington, USA.

**Keywords:** Infectious disease, Antigen-presenting cells, Dendritic cells, T cells

## Abstract

SARS-CoV-2 provokes a robust T cell response. Peptide-based studies exclude antigen processing and presentation biology, which may influence T cell detection studies. To focus on responses to whole virus and complex antigens, we used intact SARS-CoV-2 and full-length proteins with DCs to activate CD8 and CD4 T cells from convalescent people. T cell receptor (TCR) sequencing showed partial repertoire preservation after expansion. Resultant CD8 T cells recognize SARS-CoV-2–infected respiratory tract cells, and CD4 T cells detect inactivated whole viral antigen. Specificity scans with proteome-covering protein/peptide arrays show that CD8 T cells are oligospecific per subject and that CD4 T cell breadth is higher. Some CD4 T cell lines enriched using SARS-CoV-2 cross-recognize whole seasonal coronavirus (sCoV) antigens, with protein, peptide, and HLA restriction validation. Conversely, recognition of some epitopes is eliminated for SARS-CoV-2 variants, including spike (S) epitopes in the Alpha, Beta, Gamma, and Delta variant lineages.

## Introduction

The acquired immune response to SARS-CoV-2 can limit infection, as shown by protection from reinfection (1) and the efficacy of vaccines (2). While antibodies can prevent infection and disease, T cell depletion studies of convalescent or vaccinated animals strongly suggest active roles for T cells (3). Many studies have defined regions of the predicted SARS-CoV-2 proteome that can activate T cells in blood (4, 5). This work typically uses single or pooled peptides, or peptide-based reagents such as HLA multimers. Some workflows result in cell death that limits follow-up, while others, such as activation-induced marker–based (AIM-based) cell sorting (6), allow recovery of live cells for downstream work. Taken together, peptide-based studies provide a large thesaurus of reactive peptides and, in some cases, relevant HLA restricting alleles and TCR sequences. However, validation of reactivity with whole virus or complex antigens is less frequently reported.

This report used PBMC from a cohort of COVID-19 convalescent individuals (7–10) to study T cell responses to SARS-CoV-2 in the contexts of direct- and cross-presentation of complex viral antigens. Validation with proteome-covering full-length protein and peptide sets allowed definition and confirmation of individual epitopes. Expanded responder cell populations also permit detailed study of HLA restriction, functional avidity, cross-recognition of seasonal coronavirus (sCoV), and recognition of circulating SARS-CoV-2 variants including variants of concern (VOC).

Estimates of the overall magnitude of the CD4 and CD8 T cell response to SARS-CoV-2, as a percentage of circulating PBMC at times soon after recovery are around the 0.5%–1% level based on the summation of peptide reactivities (4, 11, 12). We were interested in benchmarking peptide-based estimates for SARS-CoV-2 to the levels of reactivity to whole viral antigen and, in comparing epitope specificities, determined using peptides and more complex antigens. It is established that T cells show cross-reactivity to unrelated peptide epitopes presented by a single HLA allele—for example, cross-recognition of influenza and Epstein-Barr virus (EBV) peptides with HLA-A*02:01 (13), pathogenic cross-recognition of influenza and self-epitopes (14), and potentially beneficial cross-reactivity between viruses and tumor antigens (15). The same TCR can also recognize peptides presented by divergent HLA alleles—for example, the alloreactivity of herpesvirus-specific T cells against HLA-mismatched antigen presenting cells (APC) bearing endogenous peptides (16, 17). Reactivities detected using SARS-CoV-2 peptides could include such cross-reactivity. The use of complex SARS-CoV-2 antigens to enrich responses also incorporates antigen processing and presentation, such as proteosomal cleavage, peptide transport into the endoplasmic reticulum, and trimming during HLA loading, that can be influenced by flanking sequences within proteins, as well as by potentially incorporating posttranslational modifications and cryptic ORFs absent from peptide sets (18, 19). We also studied recognition of whole SARS-CoV-2 at the effector stage. CD8 T cell assays used infected bronchial epithelial cells that were HLA matched to CD8 effector T cells, while CD4 T cell readouts used SARS-CoV-2 antigen and appropriate APC. Importantly, we also checked if T cells enriched using simpler peptide or protein antigens could recognize whole virus.

SARS-CoV-2 shares genomic structure and sequence with sCoV. Cross-reactivity with sCoV has been described (20) but is less studied in the whole virus context. SARS-CoV-2 also shows modest sequence variation, and variants and deletions emerge within-subject in immune-suppressed individuals (21), and within populations (22). In the present report, we use culture-amplified T cell responders to probe recognition of sCoV and variant SARS-CoV-2 T cell epitopes.

## Results

### Subjects and specimens.

We studied 26 specimens from 12 subjects (Supplemental Table 1; supplemental material available online with this article; https://doi.org/10.1172/jci.insight.158126DS1) with COVID-19 in spring 2020, prior to detection of VOCs in the region (23). Each subject reported SARS-CoV-2 RNA detection, positive serum or plasma anti–S domain 1 and anti–N IgG, and SARS-CoV-2 neutralizing antibody (nAb) titer ≥ 1:40 (7). Median age was 56 (range, 32–72), and sex was balanced between male and female participants. Among 4 hospitalized subjects, 3 required intensive care. Median illness duration was 16 days (range, 4–32). PBMC were obtained a median of 138 days after recovery (range, 31–256). We used several methods to enrich SARS-CoV-2–specific T cells (Supplemental Figure 1).

### PBMC T cell responses to whole SARS-CoV-2 antigen.

**Figure 1:**
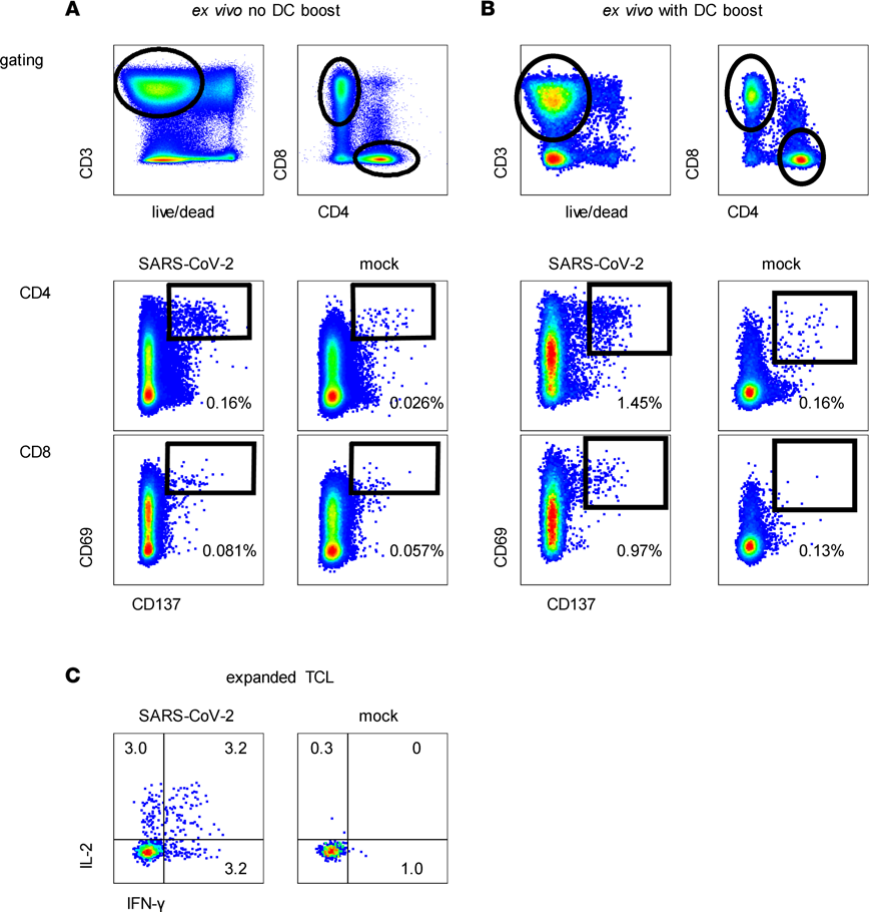
AIM detection and enrichment of SARS-CoV-2–specific T cells in response to whole virus. (**A**) PBMC from participant W003 incubated with inactivated cell-associated SARS-CoV-2 or mock antigen. Gating scheme at top. Lower panels show expression of activation markers CD137 and CD69 in response to 18-hour stimulation among CD4 or CD8 T cells. (**B**) Similar layout for subject W005, specimen 1 PBMC stimulated with autologous moDC pretreated with SARS-CoV-2 or mock antigen. Gating scheme at top. For both stimulation methods, numbers are percentages of gated T cells expressing dual activation markers. (**C**) CD69^+^/CD137^+^ CD4 T cells from the pathway in **A** were expanded and tested for reactivity with inactivated cell-associated SARS-CoV-2 or mock antigen. Gated, live, responder, CD3^+^/CD4^+^ CD8 cells are shown. Numbers are percent of cells accumulating the indicated cytokines.

SARS-CoV-2 Washington 1 (WA1) antigen was prepared from infected cells, rather than from purified virions, to include nonstructural proteins (NSPs) (24). We used dual expression of CD137 and CD69 for analytic AIM and to sort cells for expansion (Figure 1) (25). We observed a median of 0.16% (range, 0.089%–0.33%) of CD4 T cells were AIM^+^ (*n =* 5; Supplemental Table 2). Responses to mock antigen were low (median, 0.026%; range, 0.009%–0.061%). Responses to viral antigen were higher than to mock antigen (*P =* 0.0078 by pair-wise analysis); the median net response was 0.13%. Absolute and net responses to whole SARS-CoV-2 antigen in healthy donors (HD) PBMC were low, with no significant net virus-specific signal (representative data, Supplemental Figure 2A; summary, Supplemental Figure 2B). We sorted a median of 389 AIM^+^ CD4 T cells (machine counts; range, 375–2138) per specimen from the 5 COVID-19 convalescent subjects (Supplemental Table 2).

After expansion with a generalized mitogenesis protocol, T cell line (TCL) functional enrichment was measured as reactivity to whole SARS-CoV-2. Autologous PBMC used as APC were removed from analysis by gating solely on responder cells as detailed in Methods, and IFN-γ and IL-2 expression was used to enumerate SARS-CoV-2–specific CD4 T cells (8, 11) among gated live CD4-expressing responder T cells coexpressing CD3 but not CD8. We observed robust TCL enrichment of virus-reactive CD4 T cells. For example, participant W003 TCL had net 8.14% of cells responding to whole SARS-CoV-2 (Figure 1C), a 61-fold enrichment over the PBMC CD4 T cell AIM signal. Enrichment ranged from 41- to 201-fold for the 5 subjects (Supplemental Table 2).

Both monocytes in fresh PBMC and monocyte-origin DC (moDC) can process and present antigens to CD4 T cells. Higher levels of PBMC CD4 T cell activation were noted when autologous DC were used as initial APC (Figure 1B) compared with direct addition of killed virus to PBMC. Background signal for mock antigen also slightly increased (median, 0.16%; range, 0.11%–0.47%). The median net proportion of AIM^+^ CD4 T cells was 0.66% (range, 0.35%–2.43%) across 12 samples. Use of moDC allowed the sorting of a median of 11,320 activated CD4 T cells per specimen (range, 8190–19,860), from which 20% were culture expanded (Supplemental Table 2) and 80% were used for ex vivo TCR sequencing.

In addition to presentation to CD4 T cells, moDC can cross-present antigen to CD8 T cells. Using moDC, we observed specific CD8 T cell activation measured by CD69 and CD137 coexpression (Figure 1B). Negative control antigen was nonstimulatory. We did not detect PBMC CD8 T cell responses in HD (Supplemental Figure 2). As with CD4 T cells, higher net proportions of AIM^+^ CD8 T cells were noted with moDC (*n =* 12; median, 0.95%; range, 0.27%–1.62%) than without (*n =* 5; median, 0.033%; range, 0.013%–0.29%) (Supplemental Figure 2B and Supplemental Table 2). A median of 842 CD8 T cells (range, 411–2611) were expanded per specimen, while 80% of the AIM-sorted cell populations were TCR sequenced.

Two subjects were studied using moDC at 5 time points each between 32 and 256 days after recovery from COVID-19. Decreasing CD4 and CD8 AIM^+^ abundance was noted over time (Supplemental Table 2). Subject W005 had consistently higher CD4 than CD8 T cell responses, with the ratio of activated cells ranging from 1.54 to 3.15. A reciprocal pattern was seen in subject W012, with consistent CD4/CD8 ratios of AIM^+^ cells < 1.

### TCR repertoire tracking during T cell expansion.

We used the moDC workflow to present whole SARS-CoV-2 antigen (above) and analyzed AIM^+^ CD4 and CD8 T cells from 3 subjects at 2 time points each. A portion of AIM^+^ cells was directly sequenced, and a portion was sequenced after expansion, with a genomic DNA-based method (26). A median of 6.75% of CDR3 aa sequences detected ex vivo were found in expanded cultures. Reciprocally, a median of 16.75% of productive CDR3 aa sequences in the expanded cultures were detected in the corresponding ex vivo samples (Supplemental Figure 3A). CDR3 abundances showed excellent agreement for AIM^+^ cells after 1 versus 2 expansions (Supplemental Figure 3B). A possible factor contributing to repertoire differences ex vivo versus postexpansion is poor recovery of DNA from small ex vivo specimens. Among the 12 ex vivo samples, the median number of productive CDR3 gene rearrangements reported was 11.6% (range, 4.9%–21.7%) of the sorting cell counts.

### Alternative generation of polyclonal SARS-CoV-2–reactive T cells.

As an alternative approach to retain an antigen processing requirement, SARS-CoV-2 proteins were expressed in COS-7 or HeLa cells. These were harvested, inactivated, and added to PBMC without (COS-7) or with (HeLa) autologous moDC. Small increments in AIM^+^ CD4 or CD8 T cells were detectable compared with mock antigen (Supplemental Figure 4A). Finally, CD8 or CD4 T cells proliferating (Supplemental Figure 5A) to SARS-CoV-2 peptide pools were sorted and expanded.

### CD8 T cell recognition of infected cells.

**Figure 2:**
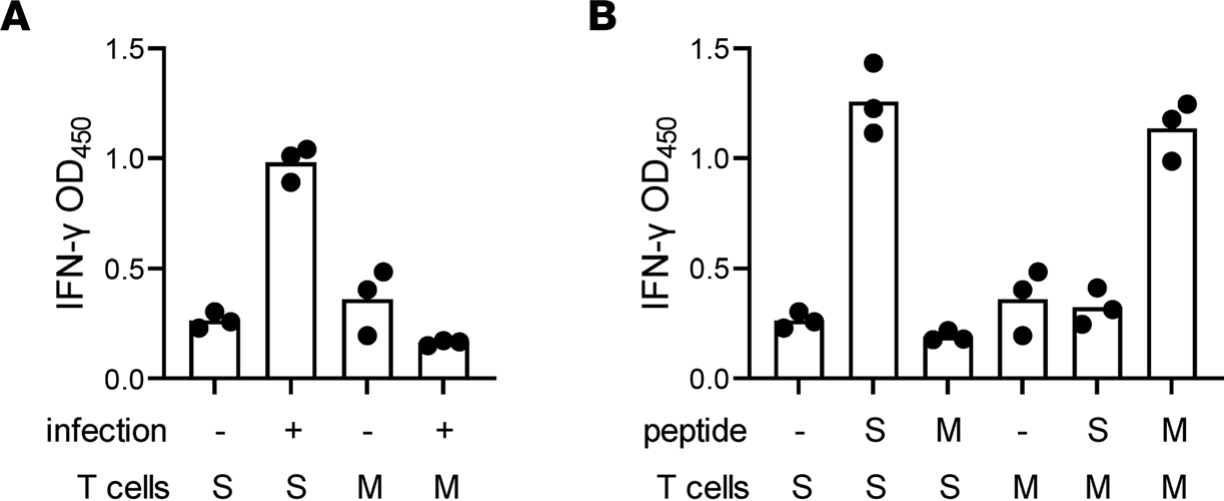
Recognition of infected respiratory epithelial cells by SARS-CoV-2–specific CD8 T cells. (**A**) HBEC3-KT-A cells with or without infection with SARS-CoV-2 were cocultivated with tetramer-enriched, HLA-A*03:01-restricted, S aa 378-386–specific CD8 T cells and activation measured by IFN-γ secretion. S, S-specific T cells; M, control MCPyV-specific CD8 T cells. (**B**) Both T cell populations specifically recognized HBEC3-KT-A cells treated with relevant viral peptide. Triplicate raw data points are shown with the bar representing the mean of triplicate. Results are representative of 3 repeat experiments.

CD8 T cell recognition typically requires HLA matching. HBEC3-KT-A are permissive for SARS-CoV-2 replication and are HLA-A*03:01^+^ (Supplemental Table 3). For effector CD8 T cells, we stimulated PBMC from HLA-A*03:01^+^ participant W003 with autologous DC loaded with S protein–expressing HeLa cells, sorted AIM^+^ CD8 T cells, expanded them, and showed they recognized S protein/HLA-A*03:01 (Supplemental Figure 6A). A reactive peptide, S aa 377–389, was found (Supplemental Figure 6B) containing the HLA-A*03:01–restricted epitope S aa 378–386 (27). A fluorescent A*03:01 S aa 378–386 tetramer allowed enrichment of specific T cells (Supplemental Figure 6C). We then examined if S protein could be processed and presented by virus-infected cells. CD8 T cells specifically recognized SARS-CoV-2–infected HBEC3-KT-A cells (Figure 2A). Specificity control HLA-A*03:01–restricted, tetramer-enriched CD8 T cells, specific for an unrelated epitope in Merkel cell polyomavirus (MCPyV) (28), recognized peptide-pulsed HBEC3-KT-A positive controls (Figure 2B) but did not recognize SARS-CoV-2–infected target cells (Figure 2A).

### CD8 T cell responses to full-length SARS-CoV-2 proteins.

**Figure 3:**
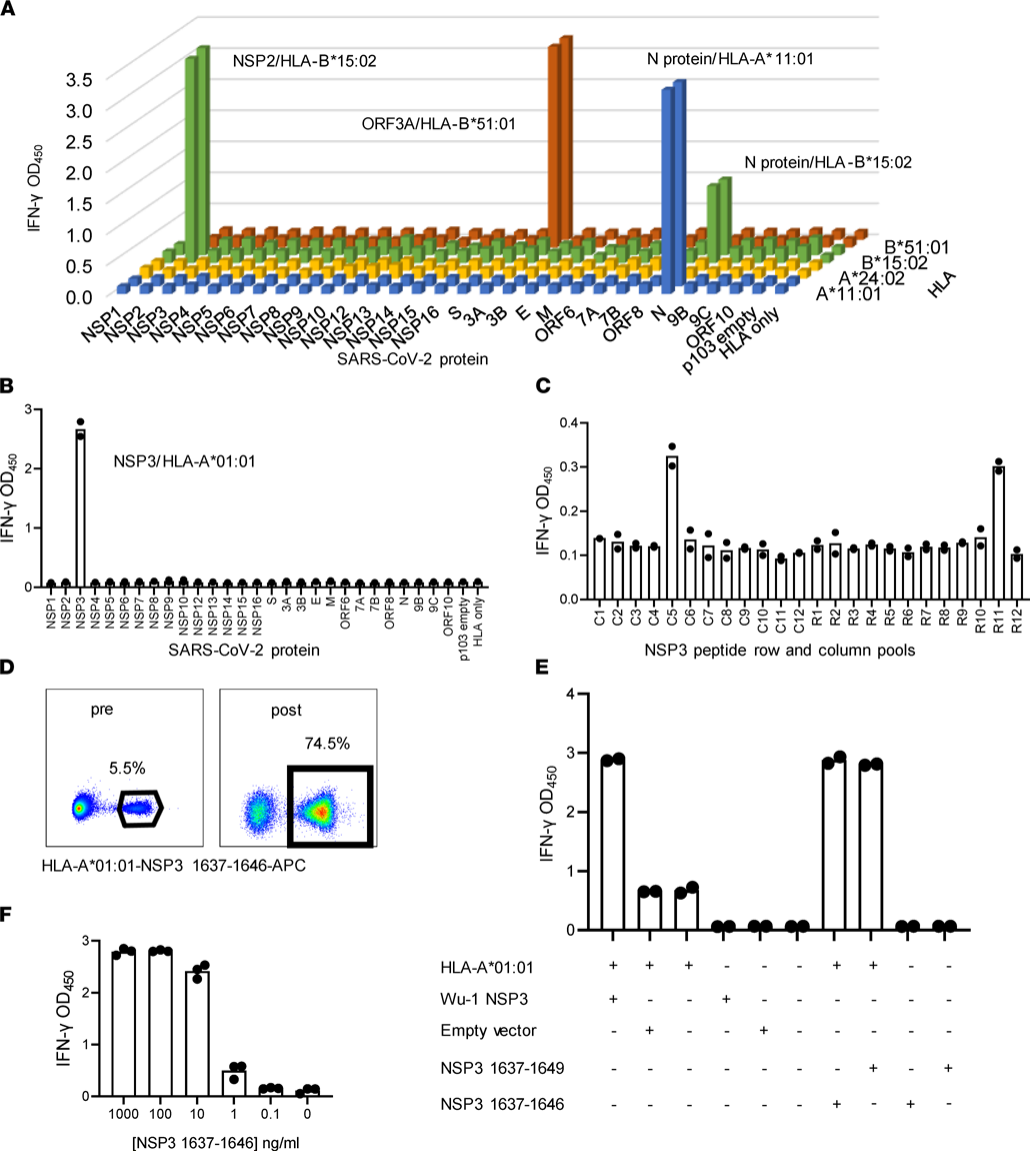
SARS-CoV-2 CD8 T cell antigens and epitopes from PBMC stimulation with whole SARS-CoV-2 antigen. (**A**) Subject W005, specimen 1 CD8 TCL were assayed with aAPC expressing each SARS-CoV-2 protein and relevant HLA-A and -B. Four reactivities were noted. (**B**) Subject W010 CD8 TCL is reactive with HLA-A*01:01 aAPC cotransfected with NSP3. For **A** and **B**, negative controls are at right. (**C**) CD8 TCL from **B** assayed against column (C) and row (R) pooled NSP3 peptides with autologous EBV-LCL as APC. (**D**) Tetramer stain of CD8 TCL before and after sorting and expansion of tetramer^+^ cells. Percentages of tetramer^+^ cells shown. (**E**) Reactivity of tetramer-enriched cells for aAPC transfected with the indicated plasmids or treated with the indicated peptides. (**F**) Dose response for HLA-A*01:01 aAPC with the indicated concentrations of NSP3 aa 1637–1646. Duplicate or triplicate IFN-γ release assays show raw data as bars (**A**) or dots for each value and means as bars (**B**, **C**, **E**, and **F**). Results are representative of 1–3 repeat experiments per panel.

**Figure 4:**
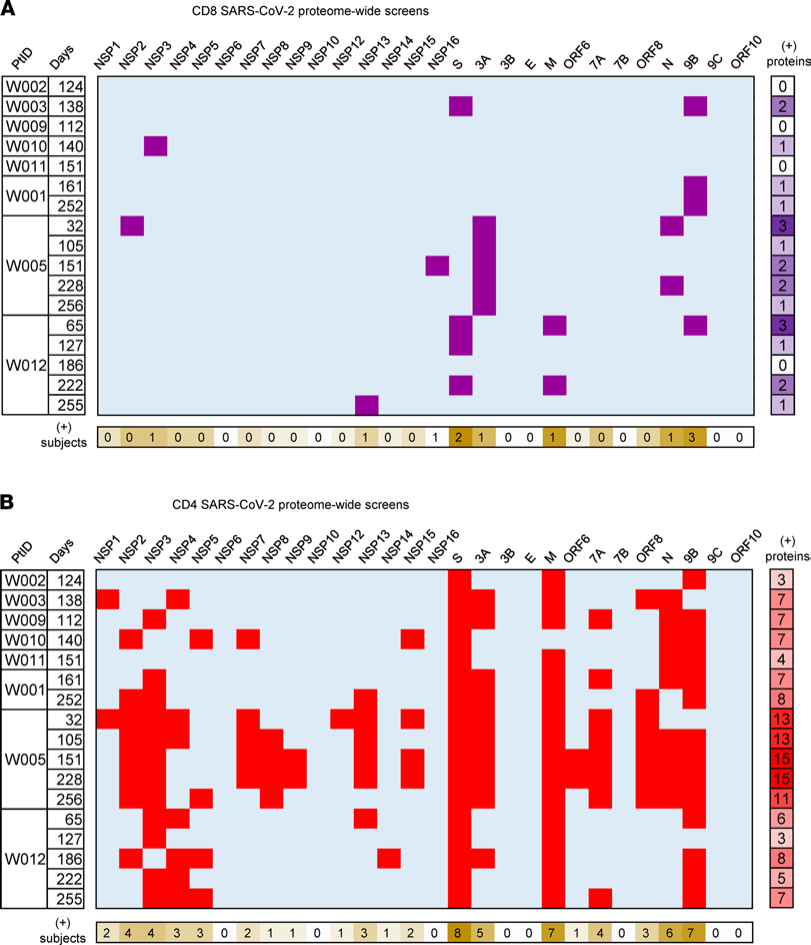
Summary of SARS-CoV-2 proteome-level T cell reactivity for PBMC after COVID-19 illness, using AIM enrichment with whole viral antigen. Rows indicate donors and days between recovery from illness and PBMC sampling. Upper 5 rows were studied without DC boosting; lower 12 rows used this procedure. Columns are individual SARS-CoV-2 proteins. (**A**) CD8 TCL scoring positive (purple). (**B**) CD4 TCL scoring positive (red). At right are number of proteins recognized and, at bottom, are number of subjects with reactivity at one or more time points. Each positive cell represents replicates, as detailed in Methods. M, membrane protein of SARS-CoV-2.

CD8 T cell target diversity was studied using whole virus stimulation followed by SARS-CoV-2 proteome-wide scans using subject-specific HLA-A and -B artificial APC (aAPC). A complex response was observed for subject W005 by incorporating DC boosting. This subject had an HLA-B*15:02–restricted response to NSP2, HLA-A*11:01– and B*15:02–restricted responses to nucleoprotein, and an HLA-B*51:01–restricted response to ORF3A (Figure 3A). Multiple proteins were positive for subject W012, while subject W001, studied using moDC, only showed responses to ORF9B (summarized in Figure 4A). Even when moDC were not used, the aAPC-proteome panels were successful for bulk AIM-enriched CD8 TCL enriched with whole virus. For example, subject W010 had a single HLA-A*01:01–restricted response detected for NSP3 (Figure 3B). This was confirmed at the peptide level (Figure 3C), allowing tetramer enrichment (Figure 3D) and use of tetramer-enriched cells confirmed as ORF and peptide reactive (Figure 3E) to determine functional avidity (Figure 3F).

### CD4 T cell responses to full-length SARS-CoV-2 proteins.

AIM-sorted, expanded CD4 TCL, enriched with or without DC boosting, were similarly tested proteome-wide. Each SARS-CoV-2 ORF or cleaved polypeptide (PP) product of ORFs PP1a and PP1ab (29) was expressed (30). Readout assays included Th1 cytokine intracellular cytokine secretion (ICS) (Supplemental Figure 7A), proliferation (Supplemental Figure 7B), and IFN-γ secretion (Supplemental Figure 7C). Across 17 specimens from 8 individuals, a median of 7 proteins were recognized per specimen (range, 3–15) (summarized in Figure 4B). The SARS-CoV-2 proteins recognized in the highest percentage of participants were S, membrane (M), nucleoprotein (N), and ORF9B (Figure 4B). Reactivity to ORF3A, ORF7A, NSP2, and NSP3 were also noted in 50% or more of participants. Because DC were not used for all specimens and few participants were studied, conclusions about antigen breadth and dominance remain preliminary. We noted that, over time, most within-participant protein-level responses were consistent. For example, participant W005 recognized NSP2, NSP13, and NSP16 at almost all time points (Figure 4B).

### CD8 T cell epitopes.

TCL initiated with diverse workflows (Supplemental Figure 1) were used. moDC–whole virus stimulation of PBMC yielded peptide epitopes (Supplemental Figure 8) to confirm ORF-level hits (Figure 3A and Figure 4A). PBMC stimulation with SARS-CoV-2 proteins with DC, followed by AIM sorting, also yielded cultures that reacted with the relevant ORF and discrete peptides (Supplemental Figure 4, A and B), as did PBMC stimulation with pooled peptides, followed by sorting of proliferated cells (Supplemental Figure 5A and Supplemental Figure 4C). Overall, integrated across CD8 TCL and eliminating redundancies, CD8 T cells reactive with 25 SARS-CoV-2 epitopes in the context of 8 HLA class I alleles were obtained (Supplemental Table 4).

### CD4 T cell epitopes.

TCL initiated with several workflows were tested to define peptide epitopes. Importantly, when TCL were started with peptide pools (as in Supplemental Figure 5A), the resultant TCL recognized whole virus lysate and full-length protein (Supplemental Figure 5B), as well as peptides (Supplemental Figure 5C), suggesting that epitopes discovered with peptides are relevant to viral infection. IFN-γ and proliferation readouts corresponded (Supplemental Figure 9), and we summarize epitopes defined with either or both readouts. Cultures initiated with complex antigen, such as from participant W001 using moDC-assisted stimulation with whole virus, yielded multiple reactive peptides (Supplemental Figure 10). Overall, CD4 T cell epitopes were detected in 13 SARS-CoV-2 proteins. Responses were diverse within-person. For specimen 1 from participant W001 (Supplemental Table 1), 55 epitopes were confirmed in 7 proteins, including 27 epitopes in S, 7 each in N and NSP3, 6 in ORF3A, 5 in M, 2 in ORF9B, and 1 in ORF7A (Supplemental Table 5). Similarly, in participant W005 studied 1 month after recovery, 65 CD4 T cell epitopes were identified in 10 proteins. Altogether, we observed 240 CD4 T cell peptide reactivities from 8 individuals (Supplemental Table 5). These correspond to 172 unique SARS-CoV-2 peptides (Supplemental Table 5). Among these, we found 70 epitopes in S protein, 26 in N protein, and 23 in M protein, with fewer in ORF3A, ORF7A, ORF8, and ORF9, as well as in NSP1, NSP2, NSP3, NSP4, NSP6, and NSP7. Peptides with CD4 T cell recognition by at least half the population studied included M aa 69–81, M aa 177–189, S aa 133–145, S aa 165–177, N aa 289–301, and N aa 349–361.

### CD4 T cell HLA restriction and functional avidity.

Studies of CD4 T cell HLA restriction and estimated functional avidity (Supplemental Figure 11) yielded HLA locus–level data on 118 of 123 (96%) peptides tested. Among these, 84 (72%) were HLA-DR restricted, 23 (20%) were HLA-DQ restricted, and 11 (8%) were HLA-DP restricted (Supplemental Table 5). HLA restriction at the locus level was generally identical if 2 participants recognized the same peptide, but there were exceptions. S protein aa 165–177 was HLA-DP restricted in 4 participants and HLA-DR restricted in 2 participants. Allele-level restriction was determined using aAPC (Supplemental Figure 11, A and B). We observed presentation by 10 distinct HLA-DRB1, -DRB3, and -DRB4 alleles (Supplemental Table 5). Some peptides in SARS-CoV-2 S, M, and N proteins were presented by both DRB1 and either DRB3 or DRB4 (Supplemental Figure 11C) or dual DRB3 alleles (not shown). Functional avidity data were available for 124 CD4 T cell epitopes (Supplemental Figure 11 and Supplemental Table 5). Responses at 1 and 10 ng/mL peptide were noted for 4 and 10 epitopes, respectively. S aa 165–177 was particularly potent, with responses at 1 or 10 ng/mL for 5 of 6 subjects.

### Recognition of sCoV.

**Figure 5:**
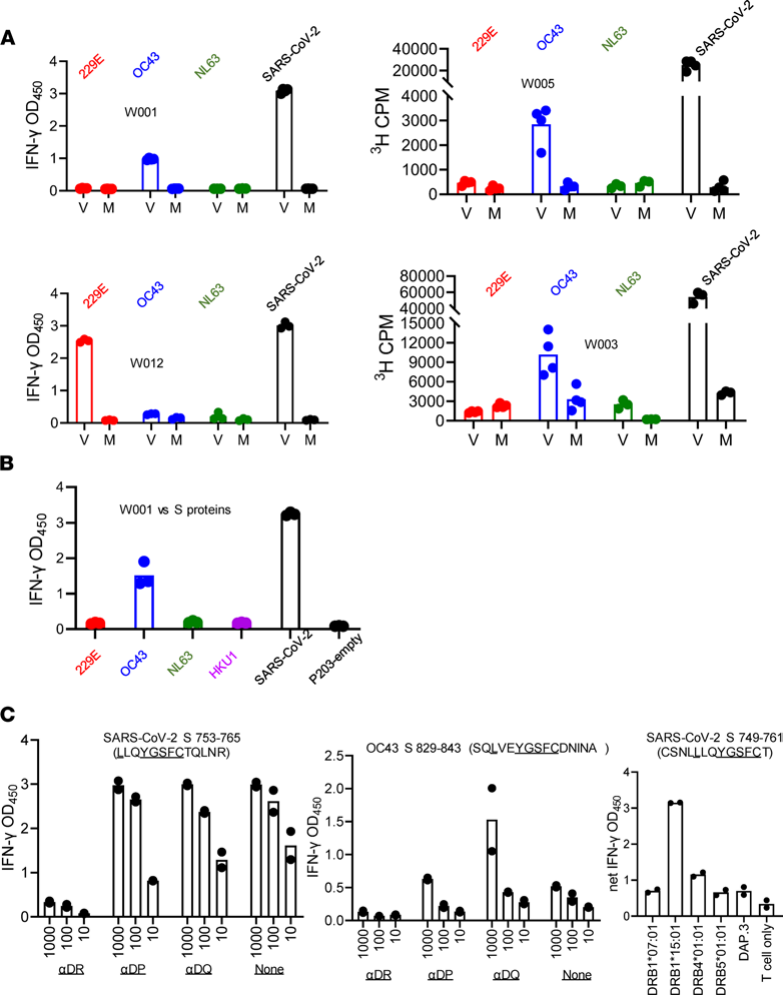
CD4 T cell coronavirus crossreactivity. (**A**) CD4 TCL from PBMC stimulated with moDC and whole SARS-CoV-2 (subjects W001, W005, W012) or PBMC stimulated with whole SARS-CoV-2 (subject W003) recognize whole SARS-CoV-2, and sCoVOC43 or 229E antigens (V), but not mock (M) antigens. (**B**) CD4 TCL from subject W001 recognizes full-length S protein from OC43 but not empty vector control. (**C**) CD4 TCL recognize homologous S peptides from SARS-CoV-2 and OC43 in an HLA-DR–restricted fashion as indicated by inhibition with locus-specific mAb. An overlapping SARS-CoV-2 peptide shows DRB1*15:01 restriction at right. Conserved aa are underlined. Duplicate or triplicate raw data and mean bars are shown. Results are representative of 1–2 repeat experiments per panel.

Cross-recognition of SARS-CoV-2 and sCoV peptide has been documented (6, 20), but less is known about recognition of complex viral antigens. We found that polyclonal CD4 TCL of 3 subjects studied, enriched from PBMC using whole SARS-CoV-2 antigen and moDC, cross-recognized either whole OC43 or whole 229E cell-associated virus (Figure 5A). Control mock-infected virus preparations were negative. An additional CD4 TCL enriched using whole SARS-CoV-2 antigen without moDC also cross-recognized OC43. For subject W001, S proteins from both viruses were antigenic (Figure 5B), as were both homologs of a peptide in S. The HLA restricting allele was established as HLA-DRB1*15:01 (Figure 5C). Cross-recognition of an HLA-DP–restricted peptide that is nearly identical in SARS-CoV-2, OC43, and HKU1 N proteins was also observed (Supplemental Figure 12).

### Recognition of SARS-CoV-2 variants.

**Figure 6:**
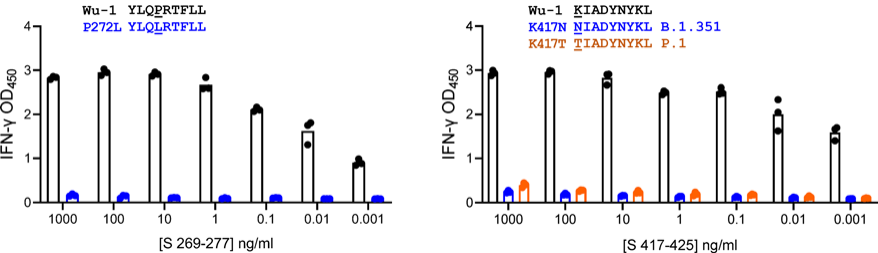
CD8 TCL recognition by SARS-CoV-2 variant peptides. aAPC transfected with HLA-A*02:01 and treated with WT but not variant peptides were recognized by polyclonal CD8 TCL lines. Lineage B.1.351 is also known as Beta and P.1 is also known as Gamma. Triplicate raw data and mean bars are shown.

To choose variants for study, we correlated SARS-CoV-2 T cell epitopes detected using Wuhan-Hu-1 (Wu-1)/WA1 reagents with variants both in early 2020 data and in early 2021 variants being monitored (VBM), concentrating on the B.1.1.7, B.1.351, and P1 lineages. Polyclonal CD8 TCL were recovered from subject W004 using an S peptide pool (Supplemental Figure 5A). Potent recognition of strain Wu-1 peptides peptides S aa 269–277 YLQ and S aa 417–425 KIA, both HLA A*02:01-restricted epitopes (27), was observed (Figure 6). There was no recognition of variants with substitutions K417N and K417T, which are present in the B.1.351 and P.1 lineages, respectively. Epitope-specific T cells enriched with tetramers (Supplemental Figure 13A) detected full-length S processed by HLA-A*02:01–transfected aAPC (Supplemental Figure 13B) but failed to recognize S K417T or the full-length SARS-CoV-2 S variant from lineage B.1.351 bearing K417T. Responses to S from B.1.1.7, which does not have an aa 417 substitution, were intact. Control CD8 T cells specific for S aa 269–277 YLQ, unchanged in these variants, were not affected (Supplemental Figure 13B). We also observed loss of CD8 TCL reactivity to SARS-CoV-2 S F490S in the HLA-A*29:02–restricted aa 489–497 epitope, to S K378N in the HLA-A*03:01–restricted aa 378–386 epitope, and to M (membrane) T175M in the HLA-A*11:01 aa 169–181 epitope (Supplemental Figure 13 and Table 1).

**Figure 7:**
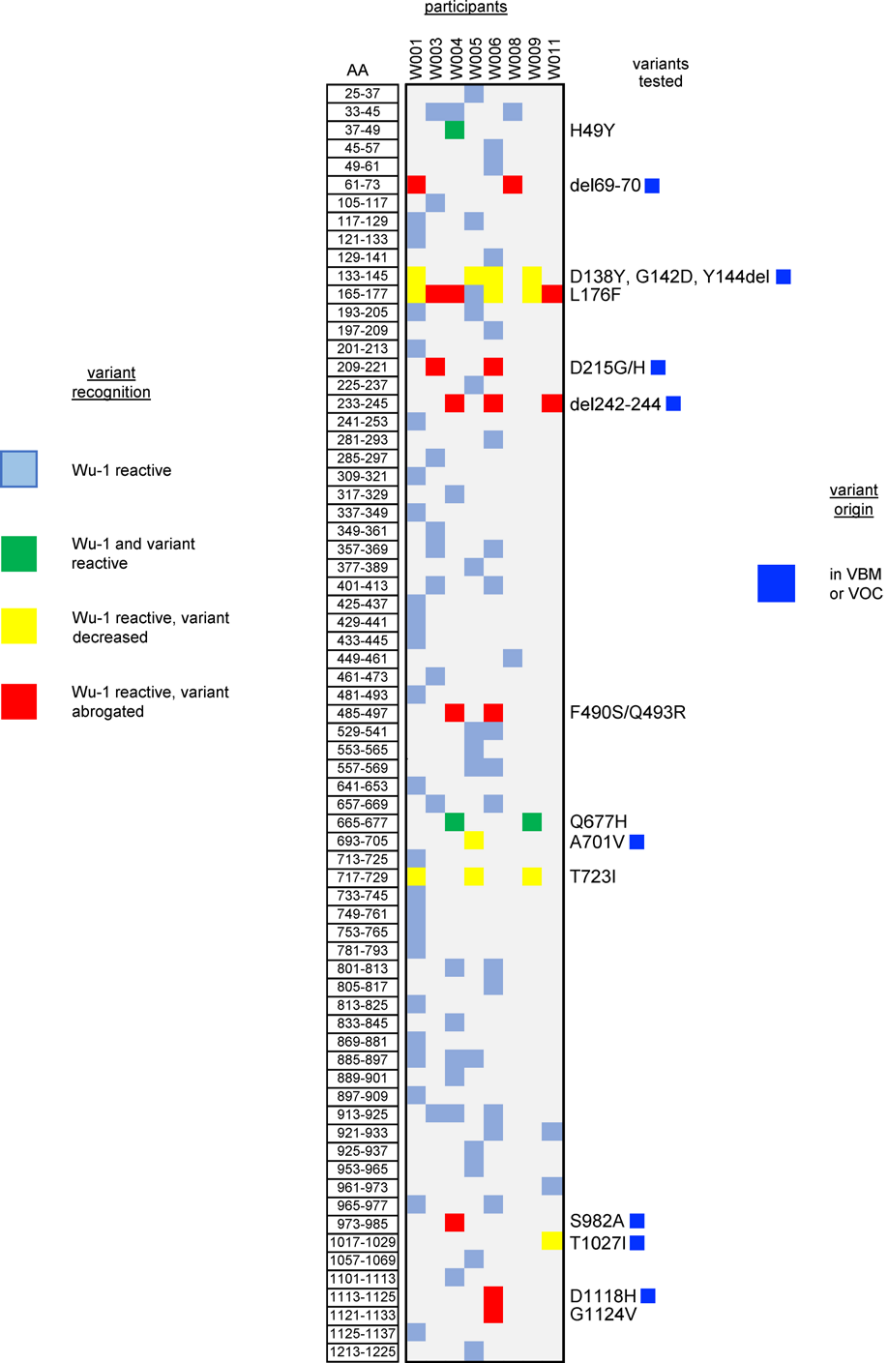
CD4 TCL recognition by SARS-CoV-2 variant peptides. Summary of recognition of S strain Wu-1 peptides and variants. Donors indicated at top. Each Wu-1 peptide recognized by 1 or more subject is numbered at left. Coordinates and aa substitutions or deletions of variants tested are listed at right. Variants in WHO VOC are indicated with blue squares. Color codes at left summarize level of recognition of variant peptide by each TCL. VBM, variants being monitored; VOC, variants of concern per US CDC October 2021. Each cell represents data from a duplicate or triplicate experiment, coded as detailed in Methods.

Variant changes could also influence CD4 T cell recognition (Supplemental Figures 14–17). In addition to loss of recognition of variant aa in B.1.1.7 (Table 1 and Figure 7) and/or P.1 lineages, we also noted strain-specific recognition of B.1.1.7-associated sequences that were already prevalent in early 2020 and not unique to VBM (Supplemental Figure 10, blue). A graphical summary of variants evaluated in S (Figure 7) shows a mixture of responses that are either preserved, partially reduced in dose-response assays, or abrogated when tested with variant peptides.

## Discussion

The T cell response to SARS-CoV-2 is functionally important (31). Studies have correlated T cell kinetics, magnitude, or phenotypes with disease in apparently immunocompetent individuals with delayed or discoordinated immunity and poor clinical outcome (8, 32). T cell responses have been linked to protection from reinfection (33). Disease states and iatrogenic treatments that decrease T cell responses can prolong live virus shedding (34, 35). Cooperation between B cells and certain lymph node T cells, such as antigen-specific CD4 T follicular helper cells (36), also suggests that T cells are involved in pathogen control. Supporting this, S-specific CD4 T cell TCR breadth and depth correlate with nAb titers in COVID-19 convalescent individuals (9). CD8 T cell depletion data in nonhuman primates indicate that these effectors contribute to vaccine-induced protection (3).

Many groups have detected responses to SARS-CoV-2 epitopes using peptide-based technologies (31), but less work has focused on T cell reactivity to complex antigens such as whole virus or full-length proteins. Both T cell priming and effector responses of memory T cells occur in vivo in the context of viral infection and/or loading of antigen into various APC, with important differences for HLA class I and II (37). In the present report, we leveraged the ability of moDC to cross-present whole viral antigen to CD8 T cells, and to present viral antigen to CD4 T cells, in order to enrich polyclonal SARS-CoV-2–specific T cells. Similar studies are rare; in one report, live virus induced subtle IFN-γ expression in convalescent T cells, but confirmation of virus specificity was not documented (38). Using established (25, 30, 39–42) readout methods, TCL created after whole virus or simpler antigen stimulation were used to study the breadth and specificity of the T cell response, reactivity to virus-infected cells, and recognition of sCoV and SARS-CoV-2 variants.

Acute COVID-19 severely effects the respiratory tract. It is likely that T cell contributions to host defense and possibly inflammatory damage occur mostly in the respiratory tract and draining lymph nodes. Cytotoxic T cells have the potential to kill SARS-CoV-2–infected cells. To date, reports of the ability of T cells to recognize SARS-CoV-2–infected cells are limited. We have begun such studies using the HBEC3-KT-A. We previously showed that HBEC3-KT-A cells are permissive for expression of SARS-CoV-2 protein and RNA after infection (43, 44). We find that S-specific CD8 T cells recognize virally infected cells. HBEC3-KT cells can differentiate into ciliated and mucus-producing goblet cells (45), and in vivo, ciliated respiratory epithelial cells have high levels of SARS-CoV-2 RNA (46). Zhang et al. reported that the SARS-CoV-2 ORF8 protein can downregulate HLA class I and limit CD8 T cell killing (47). Wagner et al. have shown recognition of SARS-CoV-2–infected lung cells overexpressing angiotensin converting enzyme 2 (ACE2) and HLA class I by TCR-transduced reporter cells (48). Many viruses encode CD8 T cell evasion functions, effects that can be selective for nontransformed, physiologic APC (49). We are currently optimizing SARS-CoV-2 infection of primary human nasal epithelial cells and pursuing CD8 recognition studies of other SARS-CoV-2 proteins.

A cytotoxic CD4 T cell phenotype has been detected during SARS-CoV-2 infection (50, 51). Upper and lower respiratory tract epithelial cells can display HLA class II for CD4 T cell recognition. Pulmonary alveolar macrophages are excellent APC for CD4 T cells, are abundant in COVID-19 pneumonia, and contain SARS-CoV-2 antigen (52). Thus, CD4 T cell recognition of whole viral antigen could be relevant to lung infection.

We studied T cell responses from relatively few individuals and did not evaluate novelty per-epitope, given the plethora of work in this area. Coverage of the entire viral proteome may, however, enable some novel insights. Mass spectrometry suggests a large spectrum in SARS-CoV-2 NSP abundance in infected cell cultures (53, 54) and in vivo (55, 56). Previous studies suggesting that viral protein abundance is associated with CD4 T cell immunodominance (39, 57) are supported by our finding of dominant responses to the abundant S and N proteins. The high population prevalence of CD4 responses to M, NSP3, and ORF3A are also in agreement with prior work (31). We also found responses to NSP2 and ORF9B in at least half the subjects studied proteome-wide. NSP2 has been described as a CD4 immunogen, while ORF9B was not included in some surveys (4, 11, 58–61). ORF9B is an alternative ORF within the nucleoprotein locus encoding an abundant 98 aa–long protein (53), localizes to mitochondria (62), elicits strong antibody responses (63), and is involved in evadging type I IFN responses (64). CD4 T cell breadth was studied in 17 samples from 8 individuals. We found a median CD4 breadth of 7 viral antigens/specimen, and we found that 6 of 8 individuals recognized 7 or more proteins. In contrast, a survey of 99 convalescent individuals with proteome-wide peptides and an ex vivo AIM approach found an average of 3.2 CD4 T cell antigenic proteins per person, with no subject recognizing over 6 proteins (4). Regarding CD4 T cell–B cell cognate help, we did not study antibody responses to each individual protein in this cohort. Previously, we showed only low responses to several NSPs in convalescent individuals, indicating that CD4 T cell and antibody responses may be somewhat independent (10).

TCL allowed tests of a large number of SARS-CoV-2 variant peptides. We found examples of abrogation or reduction of recognition of variants, some from VOC. The literature consensus is that T cell responses to infection or vaccination are poly-specific, such that failed variant epitope recognition may not be too deleterious (31). However, detailed investigations remain warranted. Tarke et al. tested PBMC from individuals infected early in the pandemic (65). While ex vivo T cell responses in convalescent PBMC measured by AIM did not differ between ancestral- and variant-derived peptides, IFN-γ ELISPOT detected an overall decrease for VOC compared with Wu-1. Agerer et al. focused the HLA-A*02:01–restricted response to the S aa 269–277 (66). The L270F variant showed decreased binding to HLA-A*02:01 and no activation of YLQ-specific CD8 T cells. L270F has been detected in 3 of > 1.4 million global sequences. In contrast, the P272L variant we investigated has been noted more than 9000 times. The aa 272 is modeled to face TCR (67) and not reduce HLA-A*02:01 binding. Based on models (67) and other epitope variants (68, 69), the S P272L and K417N or K417T variants studied herein could generate variant-specific T cells. COVID-19 vaccines mostly use Wu-1–like S antigen, such that vaccinee T cells may also be variant specific. Keeton et al. detected modest differences in CD8 T cell recognition of S between the vaccine and Delta variant (70). In another study (65), AIM responses to the B.1.351 S pool were lower compared with Wu-1 peptides. It is possible that antigenic experience from prior sCoV infections may also influence SARS-CoV-2 strain–specific responses. The precise epitope and HLA restriction information in this report can focus variant recognition studies of breakthrough infection as the pandemic evolves and as epitope-based (NCT04776317; https://clinicaltrials.gov/ct2/home) and variant-based vaccines (71) are studied.

This report adds to literature concerning cross-recognition of sCoV and SARS-CoV-2 (20, 27, 72–79). We documented that polyclonal CD4 TCL recognize OC43 and 229E whole virus, protein, and peptide, integrating antigen processing and presentation steps. The presence and sequencing of SARS-CoV-2 and sCoV infections in our study population are unknown. However, the timing of our specimen collection, and the fact that most adults have been infected with multiple sCoV, suggest the T cell cross-reactivities we detected may reflect prior sCoV infections boosted by SARS-CoV-2. Data concerning sCoV T cell cross-reactivity can be integrated with antibody-based (80, 81) and clinical studies (82) to determine if sCoV-specific immune memory modulate COVID-19 disease and to guide development of pancoronavirus vaccines.

The current study has limitations. We observed no AIM response to whole SARS-CoV-2 in HD; however, others have documented the presence of SARS-CoV-2–reactive T cells in uninfected individuals from either naive or sCoV-primed memory repertoires (20, 83). Our approaches may be better suited to memory response to SARS-CoV-2 infection. We did not directly compare whole virus restimulation with DC versus without DC for the same PBMC sample to definitively address if DC inclusion boosts AIM signals. We noted only moderate preservation of TCR sequences comparing ex vivo sorted AIM^+^ T cells and expanded cultures. Biological factors related to TCR repertoire differences could include rare clonotypes that assorted into either the ex vivo TCR sequencing or expansion fractions of the AIM^+^ cells, rather than being represented in both. Indeed, sequencing of AIM^+^ cells ex vivo shows broad and diverse responses at the TCR sequence level even to single epitopes (84). T cell programming could render some T cells refractory to expansion (85). Technical factors could also contribute, ranging from DNA extraction or PCR inefficiencies during TCR sequencing. We discounted T cell clonotypes detected ≤ 2 times, potentially reducing concurrency between ex vivo and expanded repertoires. Alternative methods such as single-cell TCR sequencing (86) to capture the TCR repertoire AIM^+^ cells ex vivo can be applied to study the virus-specific repertoire before and after expansion. The viral antigens used could have low abundance for some viral proteins in our antigen preparation, leading to underestimation of antigen breadth.

In conclusion, T cells are a functionally important component of the specific acquired immune response to SARS-CoV-2 infection and vaccination. We have shown brisk recognition of whole SARS-CoV-2 by both CD4 and CD8 T cells from convalescent individuals, provided estimates of the integrated frequency of these cells in the circulation, and used proteome-covering tools to approach within-subject antigenic breadth at the antigen and epitope levels. We found many examples of loss of recognition of epitope variants by effector T cells recovered from individuals infected early in the pandemic, and we used whole viral antigens to document coronavirus cross-reactive T cell immunity.

## Methods

### Subjects and specimens.

The COVID-19 cohort has been described (7–10). Subjects reported SARS-CoV-2 detection and COVID-19 illness. Subjects were seropositive for serum IgG for SARS-CoV-2 S and nucleoprotein (N) (ref. 7 and Supplemental Table 1). PBMC were cryopreserved. Adult HD PBMC were collected before 2019. Subjects provided informed written consent. HLA typing was by PCR amplicon sequencing at Scisco Genetics.

### Antigens.

For whole viral antigen, SARS-CoV-2 strain WA1 (87) (Genbank, MN985325.1) was cultured on Vero-E6 USAMRIID cells (gift from Ralph Baric, University of North Carolina at Chapel Hill, Chapel Hill, North Carolina, USA) at a multiplicity of infection (MOI) of 0.1 for 48 hours to 80% cytopathic effect. Cells were recovered by scraping and were frozen/thawed 3 times. Titers ranged from 4.2 × 10^7^ to 4.8 × 10^8^ pfu/mL in Vero-E6-USAMRIID plaque assay (88) before inactivation. After UV light treatment (900 mJ/cm^2^) and 3 cycles of freeze-thaw, plaque assays were negative. Vero mock antigen was prepared in parallel. Full-length SARS-CoV-2 codon–optimized molecular clones for NSP1-NSP10, NSP12-NSP16, S, ORF3A, ORF3B, E, M, N, ORF6, ORF7A, ORF7B, ORF8, N, ORF9B, and ORF9C (also known as ORF9Bwu and ORF14) from Wu-1 (Genbank NC_045512.2), and ORF10 from strain HKU-SZ-005b (Genbank MN975262.1) cloned into pDONR207 or pDONR223 (29) (Thermo Fisher Scientific) were obtained from Addgene. Gateway reactions (Thermo Fisher Scientific) shuttled inserts to vectors pDEST103 and pDEST203 for CD8 and CD4 T cell research (30). pDEST103 expresses proteins intracellularly as fusions with eGFP driven by a CMV promoter. pDEST203 expresses 6-histidine fusion proteins with a T7 promoter (30) to express antigens via in vitro transcription/translation (IVTT) (Expressway, Thermo Fisher Scientific). SARS-CoV-2 S Wu-1 with the D614G mutation, HDM_Spikedelta21_D614G (Addgene, 158762), and isogenic mutant K417N are described (89, 90) and are gifts from Jesse Bloom at the Fred Hutchinson Cancer Research Center. Additional plasmids were designed per S consensus data (22, 91), concordant with representative sequences from Global Initiative on Sharing All Influenza Data (GISAID) (92) (EPI_ISL_760400 [B.1.1.7 lineage] and EPI_ISL_700420 [B.1.351 lineage]). These were designed with the 21 aa C-terminal deletion from Wu-1 D614G and D614G/K417N and obtained from Twist in pHDM to create HDM_Spikedelta21_B.1.351 and HDM_Spikedelta21_B.1.1.7 for transient transfection. The predicted PP sequences of strains WA1 used for PBMC stimulation and Wu-1 used for most peptides differ at ORF8 aa 84.

sCoV OC43 (NR-52725), 229E (NR-52726), and NL63 (NR-470) were from BEI Resources. OC43 was cultured on VERO-E6AT cells transduced with ACE2 and transmembrane serine protease 2 (gift from Michael Diamond, Washington University, St. Louis, Missouri, USA), 229E was cultured on Huh7 cells (93), and NL63 was cultured on MK2-LLC cells (CCL-7, ATCC). Antigen was prepared by freeze-thaw and clarification (400*g*, 10 minutes, room temperature) of infected cells and was UV inactivated, with mock uninfected antigens prepared in parallel. Viral titers measured in the producer cells prior to UV were 1.6 × 10^5^ TCID_50_/mL (OC43), 2.3 × 10^6^ TCID_50_/mL (229E) and 2.0 × 10^2^ TCID_50_/mL (NL63). Antigens were tested at 1:40 (v/v) in T cell assays. S genes from OC43, 229E, NL63, and HKU1 (R619-M89-303, R619-M66-303, R619-M90-303, R619-M91-303; 166014, 166015, 166016, 166017; Addgene) were subcloned into pDEST203. IVTT-expressed antigens were tested at 1:1000.

Peptides covering SARS-CoV-2 strain Wu-1, 13 aa long overlapping by 9 aa, were ≥ 70% pure (Genscript) and dissolved (20 mg/mL) in DMSO (Thermo Fisher Scientific). Peptides in SARS-CoV-2 ORFs PP1a and PP1ab are numbered by PP position. Pools contained ≤ 54 peptides, maintaining 1 μg/mL final in-assay concentrations each, and ≤ 0.3% DMSO. Peptides containing variant aa, shorter than 13 aa, or internal to antigenic peptides were also studied. Variants surveyed included 501Y.V1 lineage B.1.1.7 (Alpha; ref. 94), B501Y.V2 lineage B.1.351 (Beta; ref. 22), 501Y.V3 P.1 lineage (Gamma; ref. 95), and lineage B.1.617.2 (Delta) (https://www.cdc.gov/coronavirus/2019-ncov/variants/variant-info.html#Interest). sCoV homologs for defined SARS-CoV-2 Wu-1 epitopes in S, N, and M proteins were determined by sequence alignment. If ≥ 1 sCoV homolog, among OC43, HKU1, 229E, and NL63, had identity at ≥ 7 aa with antigenic peptides from SARS-CoV-2 Wu-1, 13 aa homologs of the antigenic SARS-CoV-2 peptide from all 4 sCoV were assayed. In addition, OC43 S peptides, 15 aa long/11 aa overlap (EMPS-OC43-S-1, JPT) were tested as pools or single peptides at 1 μg/mL final in ≤ 0.4% DMSO.

### SARS-CoV-2–specific T cell enrichment and expansion.

For DC-AIM–based enrichment, monocytes were enriched (CD14^+^, Stemcell Technologies) and cultured in AIM-V (Stemcell Technologies) with 10 ng/mL each IL-4 and GM-CSF (R&D). moDC were collected at day 7 with EDTA/scraping and seeded at 2.5 × 10^5^/well in 48-well plates. SARS-CoV-2 antigen was loaded into moDC by adding 25 μL UV-killed antigen in 1 mL T cell medium (TCM). After 4 hours, 2 × 10^6^ autologous total T cells (negative selection, Stemcell Technologies) were added for 18 hours. Cells were stained with anti–CD3-PE (BioLegend, UCHT1), anti–CD4-APC-Cy7 (Becton Dickinson, RPA-T4), anti–CD8-FITC (Thermo Fisher Scientific, 3B5), anti–CD69-BV421 or –PE-Cy7 (BioLegend, FN50), anti–CD137-APC (Becton Dickinson, 4B4-1), and 7-actinomycin D (7-AAD). Live CD3^+^CD4^+^CD8^–^ or CD4^–^CD8^+^ cells with CD137 and CD69 expression were bulk sorted, and 20% of sorted cells were nonspecifically expanded for 2 cycles (96), with 80% saved for TCR sequencing. moDC were also used to present single viral proteins. HeLa (CCL-2, ATCC) were transfected with SARS-CoV-2 genes cloned into pDEST103 using FuGene 6 (Promega) and collected at 48 hours. Transfected cells were suspended to 1 × 10^6^ cells/mL and UV-C treated with 3600 μJ/cm^2^. moDC were collected at day 7, and 1 × 10^5^ moDC in 0.4 mL TCM were seeded into 48-well plates for 4 hours prior to adding 1 × 10^5^ UV-treated HeLa for 1 hour. In total, 1.5 × 10^6^ autologous PBMC in 0.1 mL were added at 37°C for 18 hours. AIM sorting was also used after stimulation of PBMC without moDC addition. For whole virus, UV-inactivated SARS-CoV-2 was incubated at 1:20 to 1:40 dilution with 1.5 × 10^6^ PBMC in 0.2 mL TCM in U-bottom plates for 18 hours. Mock virus negative control and 1.6 μg/mL phytohemagglutinin-P (PHA-P) positive control were included. For viral proteins, SARS-CoV-2 individual ORFs in pDEST103 were transfected into and expressed by COS-7 cells (ATCC, CRL-1651), which were lysed by triple freeze-thaw. Pooled lysates representing multiple SARS-CoV-2 proteins were added to PBMC at a 1:1 volume ratio for 18 hours.

For proliferation-based enrichment, PBMC were labeled with cell-trace violet (CTV) (Thermo Fisher Scientific) and cultured at 4 × 10^6^/well in 24-well plates in 2 mL (TCM) (25) for 5 days with SARS-CoV-2 peptides (≤54 peptides/pool, 1 μg/mL final each) covering S, M, N, NSP6, and E. After staining with anti–CD3-PE, anti–CD4-APC (Thermo Fisher Scientific, S3.5), anti–CD8-FITC, and 7-AAD for viability, live CD3^+^CTV^lo^ cells (CD4^–^CD8^+^ or CD4^+^CD8^–^) were bulk sorted (FACSAria II, Becton Dickinson) and expanded twice (96).

### Tetramer sorting.

Monomeric HLA–β2-microglobulin (HLA-β2M) complexes with UV-labile peptide (BioLegend) were used for UV peptide exchange and tetramerized with streptavidin-APC or streptavidin-PE (Becton Dickinson) per manufacturer instructions. Bulk-expanded CD8 T cells (about 1 × 10^6^) were stained in 100 μL TCM with 2 μL tetramer for 30 minutes on ice, followed by anti–CD4-APC-H7 (Becton Dickinson, RPA-T4) and anti–CD8α-FITC (Thermo Fisher Scientific, 3B5). After 7-AAD staining, live CD8^+^CD4^–^tetramer^hi^ cells were sorted, expanded (96), and cryopreserved. CD8 T cells recognizing MCPyV T antigen (T-Ag) aa 32–40/HLA-A*03:01 were tetramer sorted from Merkel cell carcinoma tumor-infiltrating lymphocytes (28).

### T cell functional assays.

CD8 TCL reactivity to proteins was measured using COS-7 aAPC cotransfected with subject-specific HLA class I cDNA and SARS-CoV-2 ORFs, or fragments of SARS-CoV-2 ORF1a/1ab (97). HLA-A and -B cDNA alleles were amplified by RT-PCR (96) or synthesized (Genscript), cloned into pcDNA3.1(–)( Thermo Fisher Scientific) (98), and sequence verified. COS-7 in 96-well flat plates were cotransfected with 100 ng/well each of HLA cDNA and SARS-CoV-2–p103 plasmids (98). After 2 days, 1 × 10^5^ CD8 TCL/well were added for 24–48 hours into 200 μL TCM, and IFN-γ was measured by ELISA (98). For peptide responses, aAPC expressed HLA cDNA only. At 2 days, 1–10 μg/mL viral peptide or DMSO was added with responder TCL, or aAPC were peptide pulsed for 1 hour at 37°C, 5% CO_2_, and washed before adding TCL. Alternatively, specificity assays used autologous EBV–lymphocyte continuous line (EBV-LCL) as APC at 2 × 10^4^ to 5 × 10^4^ cells/well in U-bottom plates, in duplicate or triplicate, with 5 × 10^5^ to 10 × 10^5^ CD8 TCL responders/well. Single or pooled peptides were added at 1 μg/mL each final in ≤ 0.3% DMSO. For a peptide to be listed as a T cell epitope, we required recognition (IFN-γ OD_450_ > 2× DMSO control) of 1 μg/mL or less of the peptide in ≥ 2 independent assays. To measure CD8 T cell recognition of SARS-CoV-2–infected cells, human bronchial epithelial cell 3 (HBEC3) immortalized with cyclin-dependent kinase 4 and human telomerase reverse transcriptase (HBEC3-KT) (99) transduced with ACE2 (43) (HBEC3-KT-A) were infected at a MOI of 2 in 6-well plates using WA1-GFP (gift from Ralph Baric, University of North Carolina, Chapel Hill, North Carolina, USA) or mock-infected, for 24 hours, harvested with Accutase (Thermo Fisher Scientific), and plated at 20,000 cells/well in U-bottom plates. CD8 T cells were added (100,000/well) to a final volume of 200 μL TCM. Supernatants after 24 hours were assayed by IFN-γ ELISA.

CD4 T cell assays for whole virus and IVTT proteins used PBMC as APC. In duplicate or triplicate U-bottom plates, 5 × 10^4^ to 10 × 10^4^ autologous PBMC and CD4 TCL were seeded in 200 μL TCM, with whole UV-treated SARS-CoV-2 or mock antigen (1:20–1:40); IVTT preparations (1:1000–1:2000) from SARS-CoV-2, empty vector, or HSV-2 gene-containing (100) negative controls; single or pooled peptides or DMSO controls; or PHA-P (1.6 μg/mL) positive controls. T cell activation was determined by IFN-γ ELISA at 1–2 days or by ^3^H thymidine incorporation proliferation assay at days 3 and 4 (101). When proliferation was measured, autologous PBMC were irradiated (3300 rad). The criteria for positivity included that, in duplicate assays, both raw count per minute (CPM) values were at least twice the average of the negative control wells containing irrelevant HSV antigens and media.

Alternatively, responses to whole virus and IVTT proteins were measured by ICS. Polyclonal CD4 TCL were CTV labeled (25). Autologous PBMC and CD4 TCL (2 × 10^5^ to 5 × 10^5^) were coincubated in 200 μL TCM in U-bottom plates with antigens or controls at concentrations listed above. Anti-CD28 (Becton Dickinson, L293) and anti-CD49d (Becton Dickinson, L25) and Brefeldin A were added (25) with analysis at 16–18 hours. Cells were stained with Near-IR live/dead (Thermo Fisher Scientific), lysed with 1× FACS lysing solution (Becton Dickinson), permeabilized with FACS Perm 2 (Becton Dickinson), and stained with anti–CD3-PE (Becton Dickinson, SK1), anti–CD4-FITC (Thermo Fisher Scientific, S3.5), anti–CD8-PerCP5.5 (BioLegend, UCHT1), anti–IFN-γ-PE-Cy7 (B27, Becton Dickson), and anti–IL-2 (MQ1-17H12, Becton Dickinson). Data were acquired with FACSCanto and cytokine expression quantified for live, CTV^+^ CD3^+^CD4^+^CD8^–^ single cells (FlowJo 10.7.1, Becton Dickinson). For ICS-based proteome screens of CD4 TCL, 2 criteria were both required to consider a protein to be positive. The ratio of the percent of IFN-γ^+^ and/or IL-2^+^ CD4 T cells with SARS-CoV-2 antigen compared with pDEST203 empty vector–derived IVTT product was > 2. For subjects W002 and W011 with high IFN-γ background for pDEST203 empty vector, only the percent of double-positive IFN-γ/IL-2 cells was used. The difference in cytokine^+^ cells between a SARS-CoV-2 protein and empty vector was > 1%.

CD4 T cell peptide analyses used autologous EBV-LCL as APC. TCL (2 × 10^4^ to 5 × 10^4^/well) and EBV-LCL (5 × 10^3^ to 20 × 10^3^/well) were coplated in U-bottom plates in duplicate or triplicate in 200 μL TCM. Peptides at 1 μg/mL of each final concentration were added as pools or singletons in ≤ 0.3% DMSO. After 1–2 days, IFN-γ was measured by ELISA with dilution if necessary. For a peptide to be reported as an epitope, ≥ 2 wells tested with 1 μg/mL peptide were required to have an IFN-γ OD_450_ at least 2 times above DMSO background in ≥ 2 assays. For proliferation assays, peptides (1 μg/mL) were tested using autologous EBV-LCL (1 × 10^4^/well, irradiated 10,000 rad) and responder TCL (5 × 10^4^/well) in duplicate. For a peptide to be reported as an epitope, both replicates had a CPM value ≥ 2-fold the average CPM of DMSO, and—in follow-up triplicate screens—then ≥ 2 of 3 CPM values were ≥ 2-fold the average CPM of negative controls. To enumerate CD4 T cell reactivities per subject, each reactive peptide was counted as a separate epitope. If 2 variant SARS-CoV-2 peptides were reactive for the same person, this was counted as 1 reactivity. Reactivities detected in ≥ 1 workflow or PBMC time point per person were counted once.

We defined CD4 TCL HLA restriction as published (40). First, serial peptide dilutions were tested in triplicate using autologous EBV-LCL as APC ± mAbs that block HLA-DR, DP, or DQ (101). To determine allele-level restriction, engineered APC expressing single HLA class II heterodimers (40) matched study subjects. Negative controls were parental to the single antigen line (SAL). SAL assays typically used 1 μg/mL peptide. Some used SAL washed after a 1-hour pulse at 37°C with peptides in titration. CD4 TCL were added, and IFN-γ was measured by ELISA. To estimate functional avidity, we set an index value of 100% to the difference between the mean IFN-γ OD_450_ values of 1 μg/mL peptide and of DMSO. A peptide concentration was scored as positive if ≥ 2 of triplicates at that concentration yielded net IFN-γ OD_450_ values (raw minus mean DMSO) of ≥ 30% index value. For selected peptides, alternative/expanded dilutions were used.

### TCR sequencing and analysis.

AIM^+^ PBMC were frozen after sorting and DNA isolated (Qiagen blood kit). DNA from culture-expanded AIM^+^ cells was isolated by Adaptive Biotechnologies. T cell receptor β (*TRB*) complementarity determining region 3 (CDR3) sequencing was performed at Adaptive Biotechnologies using Immunoseq TCRBv4b. Two replicate DNA aliquots were parallel processed, and productive TRB CDR3 gene rearrangements present in both replicates were reported. Analyses focused on functional CDR3 aa identity.

### Data availability.

T cell epitopes have been uploaded to IEDB (102) with accession nos. 1000861 and 1000866. TCR data sets are in ImmuneACCESS (Adaptive Biotechnologies).

### Statistics.

Proportions of ex vivo AIM^+^ cells were compared within-person between mock and stimulated conditions using Wilcoxon matched-pairs signed-rank test (Instat 3.10, GraphPad). Proportions of ex vivo AIM^+^ cells after stimulation were compared using Mann-Whitney *U* test. *P* values are 2-tailed. Correlation between IFN-γ and proliferation results used linear regression and default parameters (Prism 9.1.0, GraphPad). *P* < 0.05 was considered significant. To estimate the global prevalence of SARS-CoV-2 aa variants, the worldwide SARS-CoV-2 sequence data set hosted at GISAID (92) was accessed (103) using the mutation details routine, while lineages containing specific mutations were queried via Nextstrain (23), which accesses a representative subset of GISAID.

### Study approval.

This study was approved by the University of Washington IRB as study no. 00004312. Subjects provided informed written consent.

## Author contributions

Conceptualization was contributed by DMK, LJ, and XW. Data curation and bioinformatic analysis were contributed by XW, LJ, MPK, and DMK. Acquisition of funding was contributed by MG, DEG, and DMK. Investigation was contributed by LJ, XW, MPK, VLC, TYH, CLM, CWP, SMF, LAH, CG, DQT, KJL, and DEG. Data management was contributed by SS. Clinical protocol was supervised by AW and ACL. Supervision was contributed by DMK and MG. Writing was contributed by DMK, XW, MPK, LJ, and KJL.

## Supplementary Material

Supplemental data

Supplemental tables 1-6

## Figures and Tables

**Table 1:**
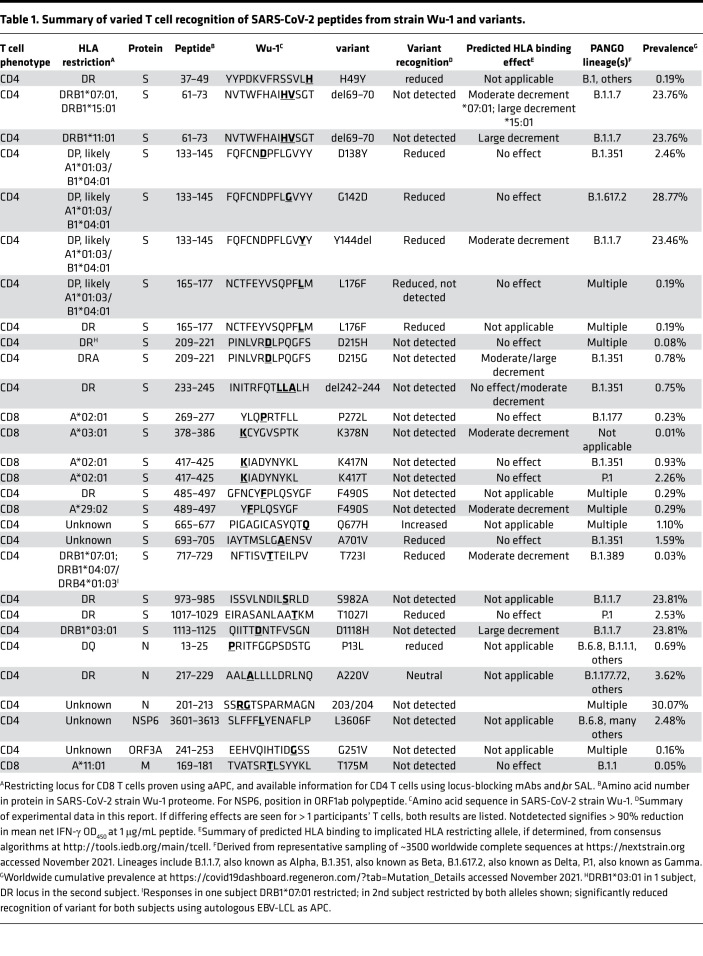
Summary of varied T cell recognition of SARS-CoV-2 peptides from strain Wu-1 and variants.

## Version 1 02/08/2022

In-Press Preview

## Version 2 03/22/2022

Electronic publication
